# Epidemiology, Clinical Data, and Management of Aseptic Abscess Syndrome: Review of Published Cases Outside France

**DOI:** 10.3390/epidemiologia6030044

**Published:** 2025-08-07

**Authors:** Gerasimos Eleftheriotis, Michaela Fragonikolaki, Chrysi Karelaki, Ergina Syrigou, Spyridon Georgiadis, Kyriaki Georgiadi, Elias Skopelitis

**Affiliations:** 1 Department of Internal Medicine, Sotiria General Hospital, 11527 Athens, Greece; mixaela.fragonikolaki@gmail.com (M.F.); ergina.syrigou.27@gmail.com (E.S.); spirgeor94@gmail.com (S.G.); sandy.georgiadi@gmail.com (K.G.); 2 First Department of Dermatology and Venereology, “Andreas Sygros” Hospital for Skin Diseases, Medical School, National & Kapodistrian University of Athens, 16121 Athens, Greece; chrysikarelaki@gmail.com; 3 Third Department of Internal Medicine, General Hospital of Nikaia-Piraeus “Agios Panteleimon”, 18454 Athens, Greece; iskopelitis@hotmail.com

**Keywords:** aseptic abscess, sterile abscess, pyoderma gangrenosum, Sweet syndrome, inflammatory bowel disease, lupus, rheumatoid arthritis, Behcet disease, Cogan syndrome, crizotinib

## Abstract

Aseptic abscess syndrome is a clinical entity that is being increasingly documented. Unfortunately, apart from the French registry, there are no other studies presenting collective data. In this review, we sought to analyze clinical and laboratory data from case reports published from the rest of the world. A total of 107 articles were found through our literature search in PubMed, Scopus, and Google, which contained 108 patients who met our eligibility criteria, including pediatric cases. The mean age at diagnosis was 39.1 years, and 54.6% of the patients were female. Cases were found affecting almost every organ, but the most common abscess locations were the spleen (51.9%), liver (35.2%), and lung (23.1%); 34.3% of the patients had multiorgan disease at diagnosis. An inflammatory syndrome was evident, with fever (79.6%), pain (66.7%), median white blood cell count of 16,200/μL, median C-reactive protein level of 15.5 mg/dL, and mean erythrocyte sedimentation rate of 79 mm/h. In total, 88.9% had an associated disease, with the most frequent being neutrophilic dermatosis (43.5%) and inflammatory bowel disease (31.5%); associated disease was inactive during abscess diagnosis in approximately one-quarter of patients. Moreover, 93.5% received corticosteroids with or without other agents, while 21.3% underwent excision surgery, which led to relapse if immunosuppressants were not concomitantly administered. No deaths were reported due to the syndrome, but 42.4% of cases that provided relevant data relapsed despite the relatively short follow-up period (median 1 year), either in the same or different organs. Combined immunomodulatory treatment, based on subgroup analysis, appeared protective against relapse in females and patients with splenic abscess or C-reactive protein >12 mg/dL (odds ratio 0.16 [95% CI 0.04–0.59]/*p* = 0.004, 0.09 [95% CI 0.01–0.62]/*p* = 0.008 and 0.23 [95% CI 0.06–0.92]/*p* = 0.03, respectively). Infection should always be the working diagnosis in patients with abscesses. However, if the infectious workup is negative, antimicrobials have failed, and no sepsis is present, then aseptic abscess syndrome should be considered; response to high-dose corticosteroids is a therapeutic criterion in almost all cases.

## 1. Introduction

Aseptic abscess syndrome (AAS) is an inflammatory disorder that was first described more than 30 years ago [1,2,3]. It is characterized by deep-seated, non-infectious collections consisting of neutrophils. As a neutrophil disease, it is considered by many investigators to be the internal/visceral variant of neutrophilic dermatoses (ND). The most common forms of ND are pyoderma gangrenosum (PG) and Sweet syndrome. In addition, AAS is also grouped among autoinflammatory disorders by some experts [4].

Due to its rarity and possibly decreased recognition by physicians, the only cohort study, to our knowledge, that includes 71 patients managed in France, was published by Trefond et al, shedding light on this relapsing disorder that many physicians are unfamiliar with [5]. All other references to this syndrome worldwide are available in the form of case reports, which demonstrate the difficulty of creating larger-scale registries and conducting clinical trials. In this review article, data from these case reports were analyzed in order to present data regarding AAS in individuals with diverse genetic backgrounds, add to the available evidence about the findings (clinical and laboratory) and management of AAS, and, hopefully, increase awareness. In addition, given the frequent relapses of AAS, we tried to investigate the effect of combined immunomodulation and baseline characteristics on the relapse rate.

## 2. Methods

### 2.1. Eligibility Criteria

In this review, we included case reports of patients with abscesses diagnosed outside France who were not associated with injections or foreign bodies and who fulfilled the criteria proposed by André et al [6], with modifications. We chose to exclude cases derived from French centers in order to avoid duplication. Data from the vast majority of these patients have already been thoroughly studied by Trefond et al in their 71-case series [5]. For this reason, we wanted to provide an analysis of data from the rest of the world.

André’s criteria:

(1) Deep abscess(es) on imaging, with a predominance of neutrophils if puncture or biopsy is performed;
(2) Negative blood cultures, serological or molecular tests for pathogens, and, in the case of abscess puncture or biopsy, negative infectious workup of pus or biopsy specimen;
(3) Antibiotic failure, if prescribed, after at least two weeks of treatment for typical pathogens and three months for mycobacteria;
(4) Fast clinical improvement observed the day after administering corticosteroids (CSs) (at least 0.5 mg/kg of prednisolone equivalent), followed by radiological improvement after one month of corticosteroids, sometimes associated with immunosuppressants.

Due to the origin of our data (case reports), we thought that with the above criteria, we would unnecessarily exclude cases that had received antimicrobials for shorter periods or had been managed successfully with other immunomodulatory treatments besides CS. In addition, some case reports did not provide clinical data from the day after CS initiation, but did include data about the improvement of clinical and inflammatory parameters, e.g., within the first five days; these aforementioned cases, however, were classified as AAS. Μoreover, AAS patients who had received non-CS immunomodulation were also included in the manuscript from the French registry [5]. Consequently, we slightly modified criteria (3) and (4) as follows:

(3) Failure of antibiotic or antimycobacterial regimens, if prescribed;
(4) Clinical improvement after administering CS or other immunomodulators or granulocyte monocyte apheresis (GMA), followed by radiological improvement and/or resolution of symptoms and inflammatory syndrome with the aforementioned approach and without receiving antimicrobials.

Furthermore, cases with granulomas in pathologic examination were excluded in order to have a more homogenous sample and reduce bias, given the nature of our data. The reason for this choice was that, although granulomas have been proposed as one of AAS’s microscopic features [5,6], many granulomas are not neutrophil-predominant but histiocyte/macrophage-predominant (and, consequently, do not fulfill André’s criteria) and may present in the context of other disorders that are not abscesses. Breast abscesses were also excluded to reduce bias due to their proximity to subcutaneous tissue, even though they can be an AAS manifestation.

### 2.2. Search Strategy

A systematic literature search was conducted using PubMed, Scopus, and Google from inception until 25 April 2025. The following search terms were used:

- PubMed and Scopus: (“aseptic” OR “sterile” OR “lupus” OR “polychondritis” OR “familial Mediterranean fever” OR “polyarteritis nodosa” OR “inflammatory bowel disease” OR “Crohn’s disease” OR “ulcerative colitis” OR “Behcet” OR “sarcoidosis” OR “pyoderma gangrenosum” OR “Sweet syndrome” OR “neutrophilic dermatosis” OR “rheumatoid arthritis” OR “Cogan”) AND “abscess”
- Google: “aseptic abscess”, “σύνδρομο άσηπτων αποστημάτων” (in Greek)

As a result, only manuscripts with at least their abstract written in English or Greek were included. The search was supplemented by screening all references and citations of the manuscripts collected from the previous search phase via ResearchGate.

### 2.3. Study Selection

**Figure 1:**
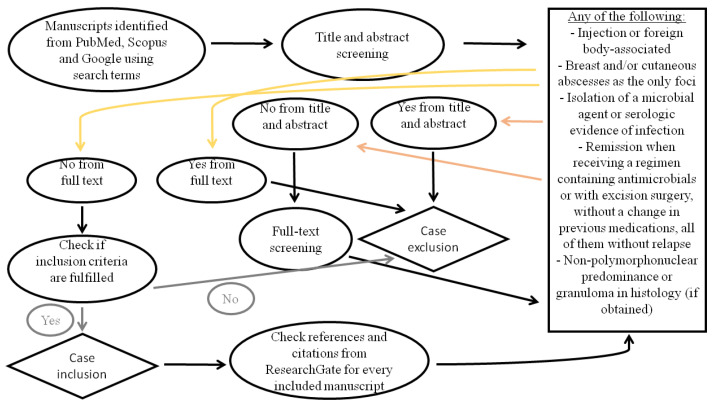
Diagram illustrating the case selection process.

Titles, abstracts, and, if needed, full texts were screened by two independent authors. Full-text screening was performed for all potentially eligible cases regarding our inclusion criteria, except those that already violated the inclusion criteria from the title and abstract. All conflicts were resolved by a third author. We also included the case report published by Weber et al. because we felt that alternative diagnoses had been excluded, even though the patient was managed successfully without immunosuppressants [7]. After all, crizotinib discontinuation was enough. A visual presentation of the case selection process is provided in Figure 1.

### 2.4. Data Extraction and Synthesis

We extracted the following information from each study using a pre-specified form (country where the case was diagnosed, gender, age, associated disorders, abscess location, clinical symptoms, laboratory data, whether an abscess puncture or biopsy was performed, follow-up data, and management). The symptom of fatigue was not extracted because we thought that it could contain reporting bias during an inflammatory syndrome. If >1 values of the same laboratory parameter were reported, then the most abnormal value was kept among those, probably attributed to AAS (e.g., a value showing a possible drug-induced hepatotoxicity was not included). In the few cases where only neutrophil count, and not whole white blood cell (WBC) count, was reported, we calculated total WBC by adding 1100 to the neutrophil count as the worst case scenario (800 lymphocytes per μL as the threshold for grade II lymphopenia according to the World Health Organization, plus 200 monocytes per μL as the threshold for monocytopenia, plus 100 eosinophils per μL as the threshold for eosinopenia) [8,9].

Composite variables were constructed as needed after data collection. Data are presented as numbers (%) for categorical variables. Continuous variables are presented as means and standard deviations (SDs) or medians and interquartile ranges (IQRs), as appropriate, and were compared using the Mann–Whitney U test for non-normally distributed variables or t-test for normally distributed variables with the Shapiro–Wilk test. The chi-square test was used to make comparisons for categorical data. All tests were two-tailed, and a *p*-value equal to or lower than 0.05 was considered significant. Τhe whole analysis was performed using SPSS version 29.0.2.0 (IBM Corp., Armonk, NY, USA). The subgroup analysis table was created with Microsoft Excel. References were constructed via EndNote™ version 20.2.1.

## 3. Results

### 3.1. Clinical and Laboratory Data

After the aforementioned screening process, 107 publications were finally selected, including 108 patients in total [7,10,11,12,13,14,15,16,17,18,19,20,21,22,23,24,25,26,27,28,29,30,31,32,33,34,35,36,37,38,39,40,41,42,43,44,45,46,47,48,49,50,51,52,53,54,55,56,57,58,59,60,61,62,63,64,65,66,67,68,69,70,71,72,73,74,75,76,77,78,79,80,81,82,83,84,85,86,87,88,89,90,91,92,93,94,95,96,97,98,99,100,101,102,103,104,105,106,107,108,109,110,111,112,113,114,115]. Another three cases may have met the inclusion criteria, but unfortunately, we did not have access to the full texts [116,117,118]. There were cases that did not fulfill the eligibility criteria, although published in the same article with included cases; this is noted in the References section. The mean age at diagnosis was 39.1 ± 19.8 years (Table 1). Patients aged <16 years old were classified as pediatric cases, and they represented 12% οf the sample; no significant differences in clinical and laboratory characteristics were observed between adults and children. Fifty-nine (54.6%) of the cases were female (Table 1). The patients were reported by Asian (38%), European (33.3%), and American (27.8%) centers, with one patient reported by an African center.

AAS diagnosis was achieved after a median of 2 months with symptoms (Table 1). Patients without an associated disorder were diagnosed later (median 5.5 vs. 2 months, *n* = 92, *p* = 0.041). Fever was present in 86 (79.6%) patients (Table 1). Normal temperature was neither associated with the presence of any associated disorder nor with abscess location. Other AAS symptoms were pain (66.7%) and weight loss (15.7%). Patients with lung abscesses presented respiratory symptoms, while mucocutaneous and articular manifestations were observed even when a relevant associated disease was absent (Table 1).

Laboratory findings, such as leukocytosis, anemia, and elevated levels of C-reactive protein (CRP) and erythrocyte sedimentation rate (ESR), were the rule; abnormal liver function tests (LFTs) were also frequent (Table 2). Νeutrophils were the predominant WBC subpopulation in all cases. In two instances without hematological malignancy, monocytosis was reported (approximately 1600/μL) [105,109]. Patients with inflammatory bowel disease (IBD) had higher CRP levels (median 20 [IQR 11.6–28.7] vs. 14.2 [IQR 7.4–24.8] mg/dL, *p* = 0.041), and patients with ND had higher hemoglobin (mean 10.6 ± 1.8 vs. 9.4 ± 2 g/dL, *p* = 0.036); no other significant associations were detected between diseases and laboratory parameters. Individuals with multiorgan abscesses at diagnosis presented higher ESR and ESR-to-upper-limit-normal ratio, adjusted for age and gender (median 3.2 [IQR 2.4–4.6] vs. 2.6 [IQR 2.2–3.5], *n* = 56, *p* = 0.048)—see Table 2.

Regarding serum ferritin, there were AAS patients with hyperferritinemia, while others had normal ferritin. The same was observed for serum albumin—some patients had hypoalbuminemia, and some did not [33,40,41,68,72,81,83,86,93,94,95,96,98,99,102,104,106,115]. Serum procalcitonin values were also available in eight cases; elevation (>0.5 ng/mL) was observed in half of them without co-existing infection.

The vast majority of patients (88.9%) had an associated disorder (Table 3). Among the rest, two patients had monoclonal gammopathy of unknown significance (MGUS). ND and IBD were the most common associated diseases (43.5% and 31.5% of total patients, respectively), while many other inflammatory and hematological diseases were reported more scarcely (Table 3). AAS without any associated disorder had no difference in age or laboratory findings. In approximately half of the cases, the diagnosis of AAS and associated disorder was concurrent. All other patients with an associated disorder, except three, had a history of an associated disease during AAS diagnosis, which was in remission in half of the cases (Table 3). Individuals who had an active associated disease during diagnosis showed no significant differences in WBC count, CRP, ΕSR, or multiorgan disease. At the time of AAS diagnosis, 8.3% (9/108) of the sample had already received immunosuppressants for their associated disorders. No comparisons could be made regarding inflammatory markers between them and the patients who did not take immunosuppressive drugs when diagnosed with AAS due to the small sample size. Moreover, we found no differences in relapse rate (*p* = 0.89), time until diagnosis (*p* = 0.38), multifocal disease (*p* = 0.5), or abscess location. However, previous use of immunosuppressants during AAS diagnosis reduced the odds of presenting with fever (44.4% vs. 82.8%, OR 0.17 [95% confidence interval {CI} 0.04–0.68], *p* = 0.006).

Regarding abscess location, the spleen (51.9% of AAS patients), liver (35.2%), and lung (23.1%) were the most frequent, but many other locations were identified (Table 1). In total, 34.3% of the cases had multiorgan disease during diagnosis; this presentation could not be linked with any associated disease. Cases with liver abscess had higher alkaline phosphatase (ALP-median 221 [IQR 158–460] vs. 97 [IQR 74–206] U/L, *p* = 0.021 [*n* = 29]). Significantly more patients with ND had pulmonary abscesses and fewer manifested hepatic abscesses (34% vs. 14.8% [*p* = 0.018] and 19.1% vs. 47.5% [*p* = 0.002], respectively).

### 3.2. Treatment and Follow-Up

Management of AAS almost universally contained CS (93.5%); one-quarter of the patients did not receive any other treatment (Table 4). Tumor necrosis factor (TNF) inhibitors were the second most prescribed drug category (26.9%), particularly anti-TNF agents blocking both soluble and transmembrane TNFα. Calcineurin inhibitors and azathioprine were used in 10.2% and 7.6% of the cases, respectively. Several other drug regimens were applied in a few patients each (Table 4). GMA was applied in 4 (3.7%) patients and used as monotherapy in one patient.

In total, 23 patients (21.3%) underwent excisional surgery; splenectomy was performed on 19 of them, while the rest had different operations (such as lung lobectomy, nephrectomy, prostatectomy, and pituitary abscess excision). All patients relapsed except three who also received immunosuppressants immediately after surgery. In cases of multiorgan abscesses, surgical procedures on one organ could not lead to remission.

In 25 (23.1%) cases, follow-up imaging was not reported or shown. The extent of the follow-up period was reported in 70 patients; median time was 12 [IQR 5.8–26.3] months (Table 4). Two patients died during follow-up, but this was not directly associated with AAS. Ιn total, 42.4% of cases for which follow-up data were available relapsed (36/85); these relapsed patients had a longer median follow-up (*p* = 0.025)—see also Table 4. As a result, cases where a more than one-year follow-up was reported had higher relapse rates (50% vs. 26.5%, *p* = 0.043). Among the 29 individuals who had abscesses in >1 organ during initial diagnosis or follow-up and experienced a relapse, 62.1% of them had a relapse in different organ(s), with or without concomitant relapse at the primary focus (Table 4). Relapse was not associated with gender, presence of fever, laboratory data, certain associated disorders, CS administration in pulse dose, multiorgan disease during diagnosis, or hepatic, pulmonary, or splenic abscesses (the latter if patients who underwent splenectomy were excluded). The presence of deep-seated lymph node abscesses was associated with more relapses (13/15 vs. 23/70, *p* < 0.001). Patients who had an active associated disorder during AAS diagnosis had fewer relapses (23.5% vs. 70.8%, *n* = 75, OR 0.13 [95% CI 0.04–0.38], *p* < 0.001), with no significant differences in laboratory parameters, multiorgan disease, or the number of immunomodulatory medications they received.

**Figure 2:**
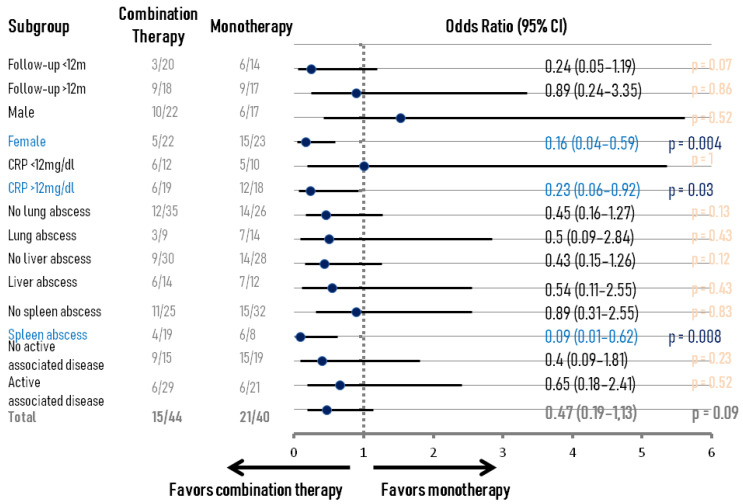
Subgroup analysis of relapse rate in patients who received monotherapy or combined immunomodulatory treatment. Patients who underwent splenectomy were excluded from the analysis for splenic abscesses, although statistical significance is obviously maintained if they were included. List of abbreviations: CI, confidence interval; CRP, C-reactive protein.

After examining the entire sample, the combination of immunomodulatory agents did not affect the relapse rate. In subgroup analysis, however, there were clinical scenarios where initial combination therapy reduced the odds of relapse. In particular, females, patients with splenic abscesses (excluding those who underwent splenectomy), and those with CRP > 12 mg/dL showed significant benefit (OR 0.16 [95% CI 0.04–0.59]/*p* = 0.004, 0.09 [95% CI 0.01–0.62]/*p* = 0.008 and 0.23 [95% CI 0.06–0.92]/*p* = 0.03, respectively)—see also Figure 2. No fewer relapses were observed in patients with a particular associated disease who received combination treatment, and results were also neutral for lymph node or multiorgan disease. Notably, no significant differences were found between males and females with AAS in splenic abscess development (*p* = 0.82) or CRP levels (*p* = 0.7), and patients with splenic abscess had similar serum CRP levels compared with the rest of the AAS cases (*p* = 0.74). Analysis of anti-TNF agents, azathioprine, and calcineurin inhibitors as part of combination therapy (almost in all cases with CS) did not reveal any advantage of a specific agent against relapse; the other medications could not be investigated due to the small sample size.

## 4. Discussion

AAS is a rare disorder that is considered a syndrome of young adults, and this is true to a large extent. In our analysis, however, one-quarter of the adults were at least 56 years old when they were diagnosed with AAS. On the other hand, we did not find any difference in age at diagnosis in cases with AAS that also had IBD (*p* = 0.082); these patients presented AAS at a younger age in a previous report [6]. In alignment with already published data, we showed that AAS is an inflammatory syndrome affecting males and females equally [5]. Apart from fever and weight loss, the rest of the symptoms predominantly depend on the abscess location. Pain site is derived from the affected organ, as well as other symptoms (e.g., cough, vomiting, seizures). Nevertheless, AAS patients can also manifest arthralgia/arthritis or skin and mucosal lesions even without an associated disorder. We found that a significant factor for an afebrile AAS presentation at diagnosis was the use of immunosuppressive drugs, which likely partially covered the clinical symptoms. Unfortunately, we could not analyze data regarding the inflammatory syndrome in these patients due to the small sample size. It would be very interesting if future studies could provide us with more data concerning this subgroup.

AAS may virtually affect any organ, but the most frequently affected organs are the spleen, liver, and lungs. Biochemical inflammatory syndrome (neutrophilia, elevated CRP and ESR, and frequently anemia and thrombocytosis) was the rule. Elevated LFTs were a common finding, while only ALP levels were associated with liver abscesses. This was not unexpected; ALP is known to be the most sensitive LFT for infectious liver abscesses as well [120].

ND and IBD were the predominant diseases associated with AAS, although other disorders are increasingly documented, along with AAS patients without any other medical history. Diagnostic workup for IBD should be offered in every patient with AAS who does not have an associated disorder [6]. Conversely, computed tomography or positron emission tomography could be considered in all patients with ND in order to exclude concomitant malignancy and AAS.

Treatment with the tyrosine kinase inhibitor crizotinib can also lead to aseptic abscess formation, in addition to renal cysts, which are a more common side effect [121]. Throughout our search, we found two more cases in which microbiological and imaging studies suggested aseptic abscesses associated with crizotinib use [122,123]. However, we excluded both patients from our analysis. The reason was the relatively strict inclusion criteria we used; one case was cured by excision surgery without relapse, and the other had a lymphocyte-predominant infiltrate in histology; therefore, they did not fulfill the criteria.

Individuals with AAS and ND more commonly had lung abscesses and less frequently had hepatic abscesses. The available evidence cannot provide explanations for this phenomenon. Variations in microbiota depending on different associated diseases could be a factor, which is under investigation at the moment in the ABSCESSBIOT study [124].

We chose not to mention MGUS as the associated disorder because it is not a disorder but a precancerous condition. It is probably not even precancerous in many individuals with inflammatory syndromes who may present transient monoclonal gammopathy due to persistent immune stimulation. Articles in the literature have reported cases of transient monoclonal gammopathy in solid organ transplant recipients or during various bacterial and viral infections, vasculitides, and autoimmune diseases, like systemic lupus erythematosus (SLE), rheumatoid arthritis, and autoimmune hepatitis, which might also be the case in AAS patients [125,126,127,128,129,130,131]. As a result, the key is the repetition of tests for the monoclonal protein after inflammatory marker normalization.

The infectious origin of an abscess should always be the initial working diagnosis, even if cultures are sterile. Aside from immunosuppression, which is a risk factor for infection, certain associated diseases like IBD increase the risk of some specific infectious abscesses (e.g., hepatic). Some clues that seem helpful for AAS diagnosis are the presence of multiorgan abscesses without concomitant emboli, after exclusion of bacteremia and infective endocarditis, and abscess spread or relapse in a different organ; however, these are not universal rules. Cases that did not have any associated disease had a later AAS diagnosis; this seems like a matter of awareness.

A patient who has abscesses and elevated serum procalcitonin should initially undergo appropriate diagnostic workup and management targeting bacterial agents. However, when this strategy fails, AAS should be in the differential diagnosis, and a CS could be initiated, especially if the patient is not septic [Sequential Organ Failure Assessment (SOFA) score < 2 from baseline]. Serum procalcitonin elevation in non-infectious diseases has already been reported. Individuals with end-stage renal disease present with mildly elevated procalcitonin. For that reason, a higher cut-off (>1.5 ng/mL) has been proposed for this population [132,133,134]. In addition, high procalcitonin values have been observed in other autoinflammatory diseases like adult-onset Still’s disease and calcium pyrophosphate deposition disease, and, more rarely, in SLE [135,136,137,138,139,140].

We observed that mortality was not an issue for AAS patients, in alignment with the available evidence [5]. However, relapses were frequent and could have a negative influence on quality of life. Sometimes relapses and remissions can also be spontaneous; this can potentially delay AAS diagnosis because such remissions can be perceived as a response to antimicrobials [61,94]. Excision surgery can indeed cause a temporary remission for AAS affecting a single organ, but relapse will occur sooner or later if immunomodulatory treatment is not administered. Sometimes, surgery is decided by physicians who are unfamiliar with the clinical entity of AAS. AAS diagnosis and management could be achieved in the least invasive fashion in virtually all cases because medical treatment is sufficient; thus, the spleen and other organs could be preserved. No response or partial response to CS was very rare (6.1%) in this study; these exceptional cases can be a diagnostic challenge.

No medication achieved remission in all patients. Some patients were diagnosed with AAS or experienced a flare while already receiving anti-TNF, azathioprine, cyclosporine, or methotrexate; trials of different medications are sometimes necessary [19,21,44,59,79,81,86,96,102]. However, biologics and azathioprine were frequently used for AAS management and have shown a relatively good safety profile. For that reason, they are reasonable options as steroid-sparing agents. Serum cytokine measurement can have a potential role in the choice of a specific biologic agent [74,92]. This choice can also be influenced by contraindications to certain drugs or an active associated disease, leading to choosing agents that cover both AAS and the associated disease according to relevant guidelines. Colchicine has been previously shown to have a protective role against relapse [5]. We were not able to investigate the effect of colchicine in this context because it was prescribed to only five patients.

In total, 61.1% of the cases received CS plus another immunomodulatory treatment at some point. However, the approach of prescribing CS in combination with another immunomodulatory treatment as a first-line choice was found to be beneficial toward relapse only for females and patients who had CRP > 12 mg/dL or splenic abscesses. The effect of combination therapy in patients with higher CRP likely indicates an increased inflammatory burden, which is more difficult to control with monotherapy. Regarding females and splenic AAS, we have no explanation for the findings concerning combination therapy. It may be a random finding without real clinical significance, or we simply do not know the answer yet; it would be very important for future studies to provide more evidence on these topics. Nevertheless, given the lack of data due to the rarity of this syndrome and the fact that combination therapy seems to be a popular tactic among treating physicians, we find it rational to initially prescribe high-dose CS plus one immunosuppressant (e.g., biologic) to patients who belong to these three aforementioned subgroups. Notably, the regimen in 60.6% of the patients who received combination immunomodulatory treatment before relapse in our review contained anti-TNF.

An unexpected protective role against AAS relapse was observed in patients with an active associated disease during AAS diagnosis. This is likely a matter of chance or a potential indication that individuals who develop AAS while already being diagnosed with another disorder and receiving immunosuppressants have a higher inflammatory burden, although this was not evident in inflammatory markers. Interestingly, the percentage of patients with an active associated disease who received a combined immunosuppressive regimen before relapse was not significantly different. More data could confirm or refute this finding in the future.

In clinically stable patients, if imaging has shown abscesses, studies for an infectious disease are negative, and antimicrobials have failed, then a CS could be empirically started. On the other hand, there are patients with Crohn’s disease or, more rarely, PG, whose imaging suggests abscesses, but whose histology reveals (extraintestinal and extracutaneous) granulomas [141,142,143,144,145,146,147]. Granulomas have been proposed as one of the AAS microscopic features [5,6]. Nevertheless, whether a neutrophilic abscess or a granuloma, both are responsive to immunosuppression; thus, no difference regarding management exists, even if a sample from the lesion cannot be obtained. The duration of treatment with immunomodulators has not been established yet. Treatment for many years could be implemented due to the relapsing nature of the syndrome, although discussion with the patient is critical, given that AAS is not fatal per se.

This study, of course, has some limitations. The retrospective nature of the primary data has possibly led to reporting bias. The main limitation, in our opinion, is the relatively short follow-up time (median 1 year), which possibly led to underestimation of the relapse rate. Projected 5-year relapse rates may be much higher, given the fact that they exceeded 60% in the French study [5]. Further follow-up could also potentially identify the presence of more associated diseases. Moreover, when an inflammatory-associated disorder is also active, it is impossible to differentiate the percentage of laboratory abnormalities attributed to AAS, although no significant differences in laboratory parameters were found in patients who had an active associated disease. Regarding the three cases for which we did not have full-text access but that potentially fulfilled the inclusion criteria, this is undeniable, although we believe that they would not drastically alter our results.

## 5. Conclusions

In summary, AAS is a rare, mostly febrile, inflammatory syndrome that can affect all sites of the body. It is clinically indistinguishable from infectious abscesses, which should be the initial working diagnosis in all cases. However, when antimicrobial regimens fail, the diagnostic workup for an infection is unrevealing, and the patient is not septic, AAS should be included in the differential diagnosis, especially when there is a history of abscess or new abscesses appear in other organs. Surgery should be avoided, if possible, because all patients relapse. Pathological examination of the excised tissue can help exclude other diagnoses. However, an abscess puncture and/or an empirical high-dose CS regimen, which should always be considered when diagnosing AAS, could provide the answer. Our study found that the combination of immunomodulatory treatments reduced relapse in females and individuals with splenic abscess or CRP > 12 mg/dL. We hope that this review will be helpful for physicians who manage patients with abscesses. Further, international-scale studies could add evidence on diagnostics and optimal management of these cases (e.g., diagnostic, prognostic, and monitoring biomarkers; effect of associated diseases or previous immunosuppressive drugs on clinical and laboratory data and prognosis; subgroups who benefit from combination treatment; steroid-sparing agent of choice; follow-up imaging protocol; and duration of treatment).

## Figures and Tables

**Table 1:** Clinical characteristics of patients.

Characteristic	*n* = 108
Mean age (years) during diagnosis–all cases	39.1 ± 19.8
Μedian age (years)–adult cases [IQR]	43 [27–56] (*n* = 95)
Μean age (years) ± SD–pediatric cases	8.8 ± 5.6 (*n* = 13) *
Female gender–no. (%)	59 (54.6)
Symptoms directly attributed to AAS–no. (%)	
Fever	86 (79.6)
Pain	72 (66.7)
Weight loss	17 (15.7)
Nausea and/or vomiting	5 (4.6)
Dyspnea	7 (6.5)
Cough	13 (12)
Hemoptysis	3 (2.8)
Mucocutaneous manifestations (*n* = 20) ^◊^	6 (30)
Others ^¶^	6 (5.6)
Median time (months) between initial symptoms and diagnosis [IQR]	2 [1–5.5] (*n* = 92)
Abscess location–no. (%)	
Spleen	56 (51.9)
Liver	38 (35.2)
Deep-seated lymph nodes	16 (14.8)
Pancreas	4 (3.7)
Kidney	8 (7.4)
Other intraabdominal	6 (5.6)
Genitalia and/or prostate	5 (4.6)
Muscle and/or deep soft tissues and/or bone–joint	14 (13)
Lung	25 (23.1)
Cardiovascular	1 (0.9)
Brain or pituitary	2 (1.9)
>1 focus during diagnosis	37 (34.3)

* Patients aged <16 years; ^◊^ only patients without IBD, neutrophilic dermatosis, BD, FMF, lupus, sarcoidosis, polyarteritis nodosa, and Cogan syndrome are reported for this manifestation. The same exclusions were made for articular symptoms, plus the patients who had rheumatoid arthritis; 3/19 of the remaining patients had articular symptoms. ^¶^ Macroscopic pyuria, seizures, and cardiac valve inflammation were reported in one patient each. Τhree patients had diarrhea/colitis after excluding patients with IBD, BD, and FMF. List of abbreviations: IQR, interquartile range; SD, standard deviation; AAS, aseptic abscess syndrome; IBD, inflammatory bowel disease; BD, Behçet disease; FMF, familial Mediterranean fever.

**Table 2:** Laboratory data.

Parameter	Value (*n*)
Median leukocytes–×10^−9^/L [IQR]	16.2 [12.9–21.4] (*n* = 74)
Mean hemoglobin ± SD (g/dL)	9.9 ± 2 (*n* = 51)
Median platelets–×10^−9^/L [IQR]	438 [281–589] (*n* = 32)
Elevated aminotransferases–no. [%] ^¶^	14/35 [40] (*n* = 35)
Median ALP–U/L [IQR]	186 [74–313] (*n* = 29)
Median γGT–U/L [IQR]	123 [32–186] (*n* = 25)
Median CRP–mg/dL [IQR]	15.5 [8.2–25] (*n* = 75)
Mean ESR ± SD (mm/h)	79 ± 30 (*n* = 56)
Median ESR to ULN ratio [IQR] ^◊^	2.8 [2.3–3.9] (*n* = 56)

^¶^ Either aspartate aminotransferase or alanine aminotransferase >40 U/L. ^◊^ ESR ULN: ½ *age (years) mm/h (+10 mm/h for females), or 20 mm/h, whichever is higher [119]. List of abbreviations: IQR, interquartile range; SD, standard deviation; ALP, alkaline phosphatase; γGT, gamma-glutamyl transferase; CRP, C-reactive protein; ESR, erythrocyte sedimentation rate; ULN, upper limit normal.

**Table 3:** Disorders associated with aseptic abscess syndrome.

Associated Disorder Data	*n* = 108
Associated disorder identified–no. (%)	96 (88.9)
Inflammatory bowel disease	34 (31.5)
Crohn’s disease	20 (18.5)
Ulcerative colitis	14 (13)
Neutrophilic dermatosis	47 (43.5)
Pyoderma gangrenosum	40 (37)
Sweet syndrome	6 (5.6)
Systemic lupus erythematosus	6 (5.6)
Rheumatoid arthritis	6 (5.6)
Behçet disease	4 (3.7)
Polyarteritis nodosa	2 (1.9)
Cogan syndrome	2 (1.9)
Sarcoidosis	2 (1.9)
Familial Mediterranean fever	1 (0.9)
OTULIN-related autoinflammatory syndrome	1 (0.9)
Weber–Christian disease	2 (1.9)
Diversion colitis	1 (0.9)
Sclerosing mesenteritis	1 (0.9)
Multiple myeloma	1 (0.9)
Acute myelogenous leukemia	1 (0.9)
Chronic myelomonocytic leukemia	1 (0.9)
Myelodysplastic syndrome	1 (0.9)
Status of associated disorder during AAS diagnosis	*n* = 95
Concomitant diagnosis	50 (52.6)
Disorder diagnosed before, active at that moment	21 (22.1)
Disorder diagnosed before, on remission at that moment	21 (22.1)
Disorder diagnosed after	3 (3.2)

List of abbreviations: AAS, aseptic abscess syndrome.

**Table 4:** Abscess diagnostics, management, and follow-up.

Parameter	*n* = 108
Abscess puncture or biopsy–no. (%)	77 (71.3)
Management–no. (%)	
CS	101 (93.5)
CS only	27 (25)
Anti-TNF	29 (26.9)
Azathioprine	19 (7.6)
Methotrexate	6 (5.6)
Hydroxychloroquine	6 (5.6)
Dapsone	5 (4.6)
Colchicine	5 (4.6)
Calcineurin inhibitors	11 (10.2)
Granulocyte–monocyte apheresis	4 (3.7)
Others *	14 (13)
Excision surgery	23 (21.3)
Max CS dosage (prednisolone equivalent)	*n* = 76
Medium (≤0.5 mg/kg/day)	11 (14.5)
High (>0.5 mg/kg/day and ≤1.5 mg/kg/day)	47 (61.8)
Pulse (>1.5 mg/kg/day)	18 (23.7)
Reason for prescribing other immunomodulator(s) (added to CS)-no. (%)	*n* = 66
Administered before AAS diagnosis for associated disorder	6 (9.1)
Administered during or after AAS diagnosis but before relapse	40 (60.6)
Administered due to the lack of response to CS during AAS diagnosis	2 (3)
Administered due to the lack of response to CS during AAS relapse	2 (3)
Administered due to AAS relapse during CS tapering	11 (16.7)
Administered due to relapse of associated disease	5 (7.6)
Relapse–no. (%)	36 (42.4) [*n* = 85]
Relapse including different organ(s) no. (%)	18 (62.1) [*n* = 29] ^¶^
Median follow-up time (months) [IQR]	12 [5.8–26.3] (*n* = 70)

* Four patients received cyclophosphamide, two received sulfasalazine, one received mycophenolate mofetil, one received leflunomide, one received intravenous immunoglobulin, while five received other biologics (anti-IL1, anti-IL6, anti-IL23, ustekinumab). ^¶^ Among patients that a relapse was reported and had multiorgan disease. List of abbreviations: CS, corticosteroids; TNF, tumor necrosis factor; AAS, aseptic abscess syndrome; IQR, interquartile range; IL, interleukin.
